# Existence theory and numerical analysis of three species prey–predator model under Mittag-Leffler power law

**DOI:** 10.1186/s13662-020-02709-7

**Published:** 2020-05-27

**Authors:** Mohammed S. Abdo, Satish K. Panchal, Kamal Shah, Thabet Abdeljawad

**Affiliations:** 1 Department of Mathematics, Hodeidah University, Al-Hodeidah, Yemen; 2 Department of Mathematics, Dr. Babasaheb Ambedkar Marathwada University, Aurangabad, India; 3 Department of Mathematics, University of Malakand, Khyber Pakhtunkhwa, Pakistan; 4 Department of Mathematics and General Sciences, Prince Sultan University, Riyadh, Saudi Arabia; 5 Department of Medical Research, China Medical University, Taichung, Taiwan; 6 Department of Computer Science and Information Engineering, Asia University, Taichung, Taiwan

**Keywords:** 26A33, 34A07, 93A30, Atangana–Baleanu and Caputo derivative, Existence and stability theory, Adams Bashforth method, Fixed point theorem

## Abstract

In this manuscript, the fractional Atangana–Baleanu–Caputo model of prey and predator is studied theoretically and numerically. The existence and Ulam–Hyers stability results are obtained by applying fixed point theory and nonlinear analysis. The approximation solutions for the considered model are discussed via the fractional Adams Bashforth method. Moreover, the behavior of the solution to the given model is explained by graphical representations through the numerical simulations. The obtained results play an important role in developing the theory of fractional analytical dynamic of many biological systems.

## Introduction

A predator–prey model is a two-component system, where one of them lives at the expense of the other. A diversity of mathematical techniques is applied at modeling a predator–prey system due to numerous factors that may affect its evolution. In this regard there have been introduced some models in [1–7] in which the first model, which regards in a specified way only substantial phenomena (gluttony and fertility), is of the type

1 $$ \textstyle\begin{cases} p^{\prime }(t)=p(t) [ a_{1}-a_{2}q(t) ] , \\ q^{\prime }(t)=q(t) [ a_{3}p(t)-a_{4} ] ,\end{cases} $$

where $p(t) $ and $q(t)$ are the number of prey, the number of predators, respectively, and $a_{1}$, $a_{2}$ and $a_{3}$ are the average of death of predators, the measurement of the tendency of prey to predation, and the predatory capability, respectively. The model (1) has a unique solution. However, the solutions of (1) are not structurally stable w.r.t. perturbation of the initial conditions. Inside the restricted scope of quadratic differential equations, those which cover competition and also predation, must be slightly more realistic. A second model with competition within preys is formulated as

2 $$ \textstyle\begin{cases} p^{\prime }(t)=p(t) [ a_{1}-a_{2}q(t)+a_{5}p(t) ] , \\ q^{\prime }(t)=q(t) [ a_{3}p(t)-a_{4} ] , \end{cases} $$

where $a_{1}a_{3}>a_{4}a_{5}$, $a_{5}>0$ describes the competition of the prey. According to biologically sensible hypotheses, there exists a unique positive solution of model (2) which is asymptotically stable.

Dai, and Zhao investigated the dynamic complexities of a predator–prey model with state dependent on impulsive influences as

3 $$ \textstyle\begin{cases} \frac{dN}{dT}=(1-\frac{N}{k})rN-\frac{a_{1}PN}{\alpha +N},\\ \frac{dP}{dT}=\frac{b_{1}a_{1}PN}{\alpha +N}+c_{1}P(1-\frac{N}{k})-d_{1}P,\\ \Delta N=-e_{1}N, \quad\quad \Delta P=e_{2}N+f. \end{cases} $$

The authors used the analogue of the Poincaré norm to obtain the existence and stability of the model (3). For details, see [8].

The following dynamic model, which addresses the case of predatory prey with disease, was analyzed by Das et al. [9]:

$$ \textstyle\begin{cases} \frac{dx}{dt}=(1-\frac{x}{k})r_{1}x+a_{1}xy-\alpha _{1}x(z+w), \\ \frac{dy}{dt}=(1-\frac{y+x}{k})r_{2}y+a_{2}xy-\alpha _{2}x(z+w)-my, \\ \frac{dz}{dt}=s_{1}\alpha _{1}xz-s_{2}\alpha _{2}yz-a_{3}zw-n_{1}z, \\ \frac{dw}{dt}=a_{3}wz-s_{3}\alpha _{1}xw-s_{4}\alpha _{2}yw-n_{2}w, \end{cases}$$

where $x(0),y(0),z(0),w(0)>0$. The authors showed that the model is globally stable on every side of the internal equilibrium point according to certain standard conditions. So, their analysis shows that the force of infection and predation average are the main parameters on the dynamics of the model.

Fractional calculus deals with differentiation and integration involving fractional order, which is advantageous over the ordinary integer order in the explanation of real-world problems, as also in the modeling of real phenomena due to characterization of memory and hereditary properties [10, 11]. Further, the integer-order derivative does not describe the dynamics between two various points. Various types of fractional-order or nonlocal derivatives were proposed in the present literature to deal with the reduction of a traditional derivative. For instance, based on a power-law, Riemann–Liouville introduced the idea of a fractional derivative. Afterwards Caputo–Fabrizio in [12] have proposed a new fractional derivative utilizing the exponential kernel. This derivative has a few problems related to the locality of the kernel. Newly, to overcome Caputo–Fabrizio’s problem, Atangana and Baleanu (AB) in [13] have proposed a new modified version of a fractional derivative with the aid of a generalized Mittag-Leffler function (MLF) as a nonsingular kernel and being nonlocal. Since the generalized MLF is used as the kernel it is guaranteed to have no singularity. Furthermore, the AB fractional derivative supplies a description of memory as discussed in [14–20].

Most of the published work describes the mathematical system of predators and prey as a problem of Cauchy type of a system of classical differential equations [21–25]. However, recently, there has been great interest in studying the behavior of the solution for some biological systems using fractional differential equations involving the Atangana–Baleanu operator by several authors for the purpose of investigating several real-world systems and modeling infectious diseases; see [26–36]. Some fractional-order models have been investigated via the new operators recently. For instance its use has been suggested for the dynamics of smoking in [32]. Along the same line, the transference model for the Ebola virus together with AB operator was studied in [31]. A fractional-order model of leptospirosis infection was considered in [26]. The dynamical behavior of coronavirus (COVID-19) epidemic infection model through the ABC derivative has been studied in [33]. Also, the existence results and analytic solutions of fractional-order dynamics of COVID-19 with ABC derivative has been obtained in [34]. There is no literature available on prey–predator fractional models with three species under the aforesaid derivative. Just some fractional models have been found in the previous years; however, they have been confined to a standard fractional derivative. Furthermore, in the presence of the mentioned derivatives, recently some fruitful results have been published in [37–39].

Due to the success of this operator in modeling the biological systems and infectious diseases, we have studied the dynamical behavior of the mathematical model which describes three prey–predator species by a nonlocal Atangana–Baleanu–Caputo (ABC) derivative operator with $0<\alpha \leq 1$ as

4 $$ \textstyle\begin{cases} {}^{\mathrm{ABC}}\mathbb{D}_{0^{+}}^{\alpha }P(t)=b_{1}P(t) (1- \frac{P(t)}{k} )-\frac{bP(t)S(t)}{a+P(t)}-r_{1}P(t)I(t), \\ {}^{\mathrm{ABC}}\mathbb{D}_{0^{+}}^{\alpha }S(t)=\frac{cbP(t)S(t)}{a+P(t)}-dS(t)I(t)-mS^{2}(t), \\ {}^{\mathrm{ABC}}\mathbb{D}_{0^{+}}^{\alpha }I(t)=-nI(t)+dI(t)+ckI(t)P(t), \end{cases} $$

with the initial conditions

5 $$ P(0)=P_{0},\qquad S(0)=S_{0},\qquad I(0)=I_{0}, $$

where ${}^{\mathrm{ABC}}\mathbb{D}_{0^{+}}^{\alpha }(\cdot )$ is the ABC fractional derivative of order *α*, $P_{0}$ is the initial population density of prey, $S_{0}$ is the initial population density of susceptible predator, and $I_{0}$ initial population density infected predator. Here *a* denotes the saturation constant whereas susceptible predators threaten the prey, *b* is a search rate of the prey across a susceptible predator, *c* is the conversion rate of the susceptible predator due to prey, and *d* is the disease transmission coefficient. The symbol *k* represents the carrying capacity of the prey population, the proportionality constant is denoted by $b_{1}$, the growth rate of the prey population is represented as $r_{1}$. In the proposed model, *m* and *n* define the death rate of sensitive predator and death rate of the infected predator, respectively. Further, we ramark that the right hand sides of our considered model (4) under ABC fractional derivtive are assumed to vanish at zero, (for details, see Theorem 3.1 in [28]).

The main aim of the paper is to demonstrate the existence, uniqueness and Ulam stability of the solution for the model (4)–(5) by using the Picard and fixed point techniques. Moreover, the numerical simulations via the fractional version of the Adams Bashforth technique to approximate the ABC fractional operator are performed. Graphical presentations are also given of the numerical results.

This paper is organized as follows: Sect. 1 presents an introduction which contains a survey of the literature. Section 2 consists of some foundational preliminaries related to fractional calculus and nonlinear analysis. The existence and Ulam stability results on a proposed model are obtained in Sects. 3, 4. The numerical solution and numerical simulations of the model at hand are presented in Sect. 5.

## Preliminaries

For the next analysis, let $0\leq t\leq T<\infty $, we define the Banach space $\varOmega =E\times E\times E$, where $E=C[0,T]$ under the norm

$$ \Vert W \Vert = \bigl\Vert ( P,S,I ) \bigr\Vert =\underset{t \in [ 0,T ] }{\max } \bigl\{ \bigl\vert P(t) \bigr\vert + \bigl\vert S(t) \bigr\vert + \bigl\vert I(t) \bigr\vert \bigr\} ,\quad P,S,I\in C [ 0,T ] . $$

### Definition 1

([13])

Let $\alpha \in (0,1] $ and $\sigma \in H^{1}(0,T)$. Then the left-sided ABC fractional derivative with the lower limit zero of order *α* for a function *σ* is defined by

$$ {}^{\mathrm{ABC}}\mathbb{D}_{0^{+}}^{\alpha }\sigma (t)= \frac{\operatorname{ABC}[\alpha ]}{1-\alpha } \int _{0}^{t}\mathbb{E}_{\alpha } \biggl( \frac{-\alpha }{\alpha -1}(t-\theta )^{\alpha } \biggr) \sigma ^{\prime }(\theta )\,d\theta , \quad t>0, $$

where $\operatorname{ABC}[\alpha ]$ is known as the normalization function which is defined as $\operatorname{ABC}[\alpha ]=\frac{\alpha }{2-\alpha }$, $0<\alpha \leq 1$ and satisfies the result $\operatorname{ABC}(0)=\operatorname{ABC}(1)=1$, and $\mathbb{E}_{\alpha }$ is called the Mittag-Leffler function defined by the series

6 $$ E_{\alpha } ( z ) =\sum_{k=0}^{\infty } \frac{z^{k}}{\varGamma ( \alpha k+1 ) }, $$

here $\operatorname{Re} ( \alpha ) >0$ and $\varGamma ( \cdot ) $ is a gamma function.

### Definition 2

([13])

Let $\alpha \in (0,1] $ and $\sigma \in L^{1}(0,T)$. Then the left-sided AB fractional integral with the lower limit zero of order *α* for a function *σ* is defined by

$$ {}^{\mathrm{AB}}\mathbb{I}_{0^{+}}^{\alpha }\sigma (t)= \frac{1-\alpha }{\operatorname{ABC}[\alpha ]}\sigma (t)+\frac{\alpha }{\operatorname{ABC}[\alpha ]}\frac{1}{\varGamma (\alpha )}\int _{0}^{t}(t-\theta )^{\alpha -1}\sigma ( \theta )\,d\theta , \quad t>0. $$

### Definition 3

([13])

The Laplace transform of ABC fractional derivative of a function $\sigma (t)$ is given by

$$ \mathcal{L} \bigl[ {}^{\mathrm{ABC}}\mathbb{D}_{0^{+}}^{\alpha }\sigma (t) \bigr] =\frac{\operatorname{ABC}[\alpha ]}{s^{\alpha } ( 1-\alpha ) +\alpha } \bigl[ s^{\alpha }\mathcal{L} \bigl[ \sigma (t) \bigr] -s^{\alpha -1} \sigma (0) \bigr] . $$

### Lemma 1

(see Proposition 3 in [40])

*The solution of the proposed problem for* $\alpha \in (0,1] $

7 $$\begin{aligned} \begin{gathered} {}^{\mathrm{ABC}}\mathbb{D}_{0^{+}}^{\alpha } \sigma (t) =\omega (t), \\ \sigma (0) =\sigma _{0} , \end{gathered} \end{aligned}$$

*is given by*

$$ \sigma (t)=\sigma _{0}+\frac{1-\alpha }{\operatorname{ABC}[\alpha ]}\omega (t)+ \frac{\alpha }{\operatorname{ABC}[\alpha ]}\frac{1}{\varGamma (\alpha )} \int _{0}^{t}(t- \theta )^{\alpha -1}\omega ( \theta )\,d\theta . $$

### Definition 4

([41])

Let ℵ be a Banach space. The operator $\varPi :\varOmega \rightarrow \varOmega $ is Lipschitzian if there exists a constant $\kappa >0$ such that

$$ \Vert \varPi \emptyset _{1}-\varPi \emptyset _{2} \Vert \leq \kappa \Vert \emptyset _{1}-\emptyset _{2} \Vert , \quad \forall \emptyset _{1},\emptyset _{2}\in \varOmega , $$

here *κ* is the Lipschitz constant for *Π*. If $\kappa <1$ we say that *Π* is a contraction.

### Theorem 1

([41])

*Let* ℵ *be a Banach space and*$\varPi :\aleph \longrightarrow \aleph $*be a contraction mapping*. *Then there exists a unique fixed point of Π*.

## Existence of solutions for the proposed model (4)–(5)

Now, we address the existence and uniqueness results of the model (4)–(5) by utilizing the fixed point technique. Let us reformulate model (4) in the appropriate form

8 $$ \textstyle\begin{cases} {}^{\mathrm{ABC}}\mathbb{D}_{0^{+}}^{\alpha }P(t)=W_{1}(t,P), \\ {}^{\mathrm{ABC}}\mathbb{D}_{0^{+}}^{\alpha }S(t)=W_{2}(t,S), \\ {}^{\mathrm{ABC}}\mathbb{D}_{0^{+}}^{\alpha }I(t)=W_{3}(t,I),\end{cases} $$

where

9 $$ \textstyle\begin{cases} W_{1}(t,P):=b_{1}P(t) (1-\frac{P(t)}{k} )- \frac{bP(t)S(t)}{a+P(t)}-r_{1}P(t)I(t), \\ W_{2}(t,S):=\frac{cbP(t)S(t)}{a+P(t)}-dS(t)I(t)-mS^{2}(t), \\ W_{3}(t,I):=-nI(t)+dI(t)+ckI(t)P(t). \end{cases} $$

Utilizing Lemma 1, the model (8) can be turned to the fractional integral equation in the sense of AB fractional integral as follows:

10 $$ \textstyle\begin{cases} P(t)-P(0)=\frac{1-\alpha }{\operatorname{ABC}[\alpha ]}W_{1}(t,P)+ \frac{\alpha }{\operatorname{ABC}[\alpha ]}\frac{1}{\varGamma (\alpha )}\int _{0}^{t}(t-\theta )^{ \alpha -1}W_{1}(\theta ,P)\,d\theta , \\ S(t)-S(0)=\frac{1-\alpha }{\operatorname{ABC}[\alpha ]}W_{2}(t,S)+ \frac{\alpha }{\operatorname{ABC}[\alpha ]}\frac{1}{\varGamma (\alpha )}\int _{0}^{t}(t-\theta )^{ \alpha -1}W_{2}(\theta ,S)\,d\theta , \\ I(t)-I(0)=\frac{1-\alpha }{\operatorname{ABC}[\alpha ]}W_{3}(t,I)+ \frac{\alpha }{\operatorname{ABC}[\alpha ]}\frac{1}{\varGamma (\alpha )}\int _{0}^{t}(t-\theta )^{ \alpha -1}W_{3}(\theta ,I)\,d\theta .\end{cases} $$

### Theorem 2

*The kernels*$W_{\ell }$ ($\ell =1,2,3$) *agree with the contraction and Lipchitz conditions if there exists a constant*$L_{\ell }$*such that*$0\leq L_{\ell }<1$, $\ell =1,2,3$.

### Proof 1

For $W_{1}$, let *P* and $P^{\ast }$ be two functions, then we have

$$\begin{aligned}& \bigl\Vert W_{1}(t,P)-W_{1} \bigl(t,P^{\ast } \bigr) \bigr\Vert \\& \quad = \bigl\Vert b_{1} \bigl( P-P^{\ast } \bigr) - \bigl( P^{2}-P^{ \ast 2} \bigr) - \bigl( P-P^{\ast } \bigr) S-r_{1} \bigl( P-P^{ \ast } \bigr) I \bigr\Vert \\& \quad \leq \biggl( b_{1}+\frac{b_{1}}{k} \bigl\Vert P+P^{\ast } \bigr\Vert +ab \Vert S \Vert +r_{1} \Vert I \Vert \biggr) \bigl\Vert P-P^{\ast } \bigr\Vert \\& \quad \leq \biggl( b_{1}+\frac{b_{1}}{k} \bigl( A_{1}+A_{1}^{\ast } \bigr) +abC_{1}+r_{1}D_{1} \biggr) \bigl\Vert P-P^{\ast } \bigr\Vert \\& \quad = L_{1} \bigl\Vert P-P^{\ast } \bigr\Vert , \end{aligned}$$

where $L_{1}:= ( b_{1}+\frac{b_{1}}{k} ( A_{1}+A_{1}^{\ast } ) +abC_{1}+r_{1}D_{1} ) $, and $\Vert P \Vert $, $\Vert P^{\ast } \Vert $, $\Vert S \Vert $, $\Vert I \Vert $are functions bounded by the constants $A_{1}$, $A_{1}^{\ast }$, $C_{1}$, $D_{1}$, respectively. Consequently

11 $$ \bigl\Vert W_{1}(t,P)-W_{1} \bigl(t,P^{\ast } \bigr) \bigr\Vert \leq L_{1} \bigl\Vert P-P^{\ast } \bigr\Vert . $$

Obviously, the Lipschitz condition is verified for $W_{1}$. Besides, $W_{1}$ leads to a contraction due to $0\leq L_{1}<1$. Likewise, we can show that $W_{2}$ and $W_{3}$ admit the contraction and Lipschitz condition, i.e.,

12 $$\begin{aligned}& \bigl\Vert W_{2}(t,S)-W_{2} \bigl(t,S^{\ast } \bigr) \bigr\Vert \leq L_{2} \bigl\Vert S-S^{\ast } \bigr\Vert , \end{aligned}$$

13 $$\begin{aligned}& \bigl\Vert W_{3}(t,I)-W_{3} \bigl(t,I^{\ast } \bigr) \bigr\Vert \leq L_{3} \bigl\Vert I-I^{\ast } \bigr\Vert , \end{aligned}$$

where $L_{2}:= ( \frac{cb}{a+A_{1}}A_{1}+dD_{1}+m ( C_{1}+C_{1}^{ \ast } ) ) $ and $L_{3}:= ( n+d+ckA_{1} ) $.

### Theorem 3

*Assume that the conditions* (11)*–*(13) *hold*. *If*

$$ \varLambda _{\ell }:= \biggl[ \frac{1-\alpha }{\operatorname{ABC}[\alpha ]}+ \frac{T^{\alpha }}{\operatorname{ABC}[\alpha ]\varGamma (\alpha )} \biggr] L_{\ell }< 1,\quad \textit{for } \ell =1,2,3. $$

*Then the solution of the fractional model given in* (4)*–*(5) *exists and is unique*.

### Proof 2

The initial conditions and the recurrence form of the model (10) are, respectively,

$$ P(0)=P_{0}(t),\qquad S(0)=S_{0}(t),\qquad I(0)=I_{0}(t), $$

and

14 $$ \textstyle\begin{cases} P_{n}(t)=\frac{1-\alpha }{\operatorname{ABC}[\alpha ]}W_{1}(t,P_{n-1})+ \frac{\alpha }{\operatorname{ABC}[\alpha ]}\frac{1}{\varGamma (\alpha )}\int _{0}^{t}(t-\theta )^{ \alpha -1}W_{1}(\theta ,P_{n-1})\,d\theta , \\ S_{n}(t)=\frac{1-\alpha }{\operatorname{ABC}[\alpha ]}W_{2}(t,S_{n-1})+ \frac{\alpha }{\operatorname{ABC}[\alpha ]}\frac{1}{\varGamma (\alpha )}\int _{0}^{t}(t-\theta )^{ \alpha -1}W_{2}(\theta ,S_{n-1})\,d\theta , \\ I_{n}(t)=\frac{1-\alpha }{\operatorname{ABC}[\alpha ]}W_{3}(t,I_{n-1})+ \frac{\alpha }{\operatorname{ABC}[\alpha ]}\frac{1}{\varGamma (\alpha )}\int _{0}^{t}(t-\theta )^{ \alpha -1}W_{3}(\theta ,I_{n-1})\,d\theta .\end{cases} $$

The successive difference between the terms is defined as

15 $$ \textstyle\begin{cases} \varPhi _{1n}(t)=P_{n}(t)-P_{n-1}(t)=\frac{1-\alpha }{\operatorname{ABC}[\alpha ]} [ W_{1}(t,P_{n-1})-W_{1}(t,P_{n-2}) ] \\\hphantom{\varPhi _{1n}(t)}\quad {} +\frac{\alpha }{\operatorname{ABC}[\alpha ]}\frac{1}{\varGamma (\alpha )}\int _{0}^{t}(t-\theta )^{\alpha -1} [ W_{1}(\theta ,P_{n-1})-W_{1}( \theta ,P_{n-2}) ] \,d\theta , \\ \varPhi _{2n}(t)=S_{n}(t)-S_{n-1}(t)=\frac{1-\alpha }{\operatorname{ABC}[\alpha ]} [ W_{1}(t,S_{n-1})-W_{1}(t,S_{n-2}) ] \\ \hphantom{\varPhi _{2n}(t)}\quad {} +\frac{\alpha }{\operatorname{ABC}[\alpha ]}\frac{1}{\varGamma (\alpha )}\int _{0}^{t}(t-\theta )^{\alpha -1} [ W_{1}(\theta ,S_{n-1})-W_{1}( \theta ,S_{n-2}) ] \,d\theta , \\ \varPhi _{3n}(t)=I_{n}(t)-I_{n-1}(t)=\frac{1-\alpha }{\operatorname{ABC}[\alpha ]} [ W_{1}(t,I_{n-1})-W_{1}(t,I_{n-2}) ] \\ \hphantom{\varPhi _{3n}(t)}\quad {} +\frac{\alpha }{\operatorname{ABC}[\alpha ]}\frac{1}{\varGamma (\alpha )}\int _{0}^{t}(t-\theta )^{\alpha -1} [ W_{1}(\theta ,I_{n-1})-W_{1}( \theta ,I_{n-2}) ] \,d\theta .\end{cases} $$

Clearly

16 $$ \textstyle\begin{cases} P_{n}(t)=\sum_{\ell =1}^{n}\varPhi _{1\ell }(t), \\ S_{n}(t)=\sum_{\ell =1}^{n}\varPhi _{2\ell }(t), \\ I_{n}(t)=\sum_{\ell =1}^{n}\varPhi _{3\ell }(t).\end{cases} $$

Taking the norm of Eqs. (15), it follows from the conditions (11)–(13) that

17 $$ \textstyle\begin{cases} \Vert \varPhi _{1n}(t) \Vert \leq \frac{1-\alpha }{\operatorname{ABC}[\alpha ]}L_{1} \Vert \varPhi _{1(n-1)}(t) \Vert + \frac{\alpha }{\operatorname{ABC}[\alpha ]}\frac{L_{1}}{\varGamma (\alpha )}\int _{0}^{t}(t-\theta )^{\alpha -1} \Vert \varPhi _{1(n-1)}(\theta ) \Vert \,d\theta , \\ \Vert \varPhi _{2n}(t) \Vert \leq \frac{1-\alpha }{\operatorname{ABC}[\alpha ]}L_{2} \Vert \varPhi _{2(n-1)}(t) \Vert + \frac{\alpha }{\operatorname{ABC}[\alpha ]}\frac{L_{2}}{\varGamma (\alpha )}\int _{0}^{t}(t-\theta )^{\alpha -1} \Vert \varPhi _{2(n-1)}(\theta ) \Vert \,d\theta , \\ \Vert \varPhi _{3n}(t) \Vert \leq \frac{1-\alpha }{\operatorname{ABC}[\alpha ]}L_{3} \Vert \varPhi _{3(n-1)}(t) \Vert + \frac{\alpha }{\operatorname{ABC}[\alpha ]}\frac{L_{3}}{\varGamma (\alpha )}\int _{0}^{t}(t-\theta )^{\alpha -1} \Vert \varPhi _{3(n-1)}(\theta ) \Vert \,d\theta .\end{cases} $$

Let us consider *P*, *S* and *I* as bounded functions that comply with the Lipschitz condition. It follows from Eqs. (16) and (17) that

18 $$ \textstyle\begin{cases} \Vert \varPhi _{1\ell }(t) \Vert \leq \Vert P_{n}(0) \Vert [ \frac{1-\alpha }{\operatorname{ABC}[\alpha ]}L_{1}+ \frac{T^{\alpha }}{\operatorname{ABC}[\alpha ]\varGamma (\alpha )}L_{1} ] ^{n}, \\ \Vert \varPhi _{2\ell }(t) \Vert \leq \Vert S_{n}(0) \Vert [ \frac{1-\alpha }{\operatorname{ABC}[\alpha ]}L_{2}+ \frac{T^{\alpha }}{\operatorname{ABC}[\alpha ]\varGamma (\alpha )}L_{2} ] ^{n}, \\ \Vert \varPhi _{3\ell }(t) \Vert \leq \Vert I_{n}(0) \Vert [ \frac{1-\alpha }{\operatorname{ABC}[\alpha ]}L_{3}+ \frac{T^{\alpha }}{\operatorname{ABC}[\alpha ]\varGamma (\alpha )}L_{3} ] ^{n}.\end{cases} $$

This shows the existence for the solutions. Moreover, to prove that Eqs. (18) are solutions for the model (4)–(5), we consider

$$ \textstyle\begin{cases} P(t)-P(0)=P_{n}(t)-M_{1n}(t), \\ S(t)-S(0)=S_{n}(t)-M_{2n}(t), \\ I(t)-I(0)=I_{n}(t)-M_{3n}(t).\end{cases}$$

Now, we consider the conditions

$$\begin{aligned} \bigl\Vert M_{1n}(t) \bigr\Vert \leq & \biggl\Vert \frac{1-\alpha }{\operatorname{ABC}[\alpha ]} \bigl[ W_{1}(t,P)-W_{1}(t,P_{n-1}) \bigr] \\ &{} +\frac{\alpha }{\operatorname{ABC}[\alpha ]}\frac{1}{\varGamma (\alpha )}\int _{0}^{t}(t-\theta )^{\alpha -1} \bigl[ W_{1}(\theta ,P)-W_{1}( \theta ,P_{n-1}) \bigr] \,d \theta \biggr\Vert \\ \leq &\frac{1-\alpha }{\operatorname{ABC}[\alpha ]}L_{1} \Vert P-P_{n-1} \Vert +\frac{T^{\alpha }}{\operatorname{ABC}[\alpha ]\varGamma (\alpha )}L_{1} \Vert P-P_{n-1} \Vert . \end{aligned}$$

On using recessive techniques, we get

$$ \bigl\Vert M_{1n}(t) \bigr\Vert \leq \biggl( \frac{1-\alpha }{\operatorname{ABC}[\alpha ]}+\frac{t_{0}^{\alpha }}{\operatorname{ABC}[\alpha ]\varGamma (\alpha )} \biggr) ^{n+1}L_{1}^{n+1}. $$

As $n\rightarrow \infty $, $\Vert M_{1n}(t) \Vert \rightarrow 0$. In a similar way, we conclude that $\Vert M_{2n}(t) \Vert $ and $\Vert M_{3n}(t) \Vert $ tends to 0.

Next, we address the uniqueness of the solution to the proposed mode (4)–(5). To this end, let $P^{\ast }(t)$, $S^{\ast }(t)$ and $I^{\ast }(t)$ be other solutions. Then

$$\begin{aligned} \bigl\Vert P(t)-P^{\ast }(t) \bigr\Vert \leq &\frac{1-\alpha }{\operatorname{ABC}[\alpha ]} \bigl\Vert W_{1}(t,P)-W_{1} \bigl(t,P^{\ast } \bigr) \bigr\Vert \\ & {} +\frac{\alpha }{\operatorname{ABC}[\alpha ]}\frac{1}{\varGamma (\alpha )}\int _{0}^{t}(t-\theta )^{\alpha -1} \bigl\Vert W_{1}(\theta ,P)-W_{1} \bigl( \theta ,P^{\ast } \bigr) \bigr\Vert \,d\theta \\ \leq & \biggl( \frac{1-\alpha }{\operatorname{ABC}[\alpha ]}+ \frac{t^{\alpha }}{\operatorname{ABC}[\alpha ]\varGamma (\alpha )} \biggr) L_{1} \bigl\Vert P(t)-P^{\ast }(t) \bigr\Vert . \end{aligned}$$

It means that

$$ \bigl\Vert P(t)-P^{\ast }(t) \bigr\Vert \biggl( 1- \frac{1-\alpha }{\operatorname{ABC}[\alpha ]}-\frac{t^{\alpha }}{\operatorname{ABC}[\alpha ]\varGamma (\alpha )} \biggr) L_{1}\leq 0. $$

From our hypothesis

$$ \biggl( 1-\frac{1-\alpha }{\operatorname{ABC}[\alpha ]}- \frac{T^{\alpha }}{\operatorname{ABC}[\alpha ]\varGamma (\alpha )} \biggr) L_{1}\geq 0. $$

It follows that $P(t)-P^{\ast }(t)=0$. Likewise, we conclude that $S(t)-S^{\ast }(t)=0$ and $I(t)-I^{\ast }(t)=0$.

## Ulam–Hyers stability

For the notion of Ulam stability, see [42, 43]. The aforesaid stability has been scrutinized for classical fractional derivatives in many of the research articles; we refer to some of them like [44–47]. Additionally, since stability is a prerequisite in respect of approximate solution, we endeavor on Ulam type stability for the model (4) via using nonlinear functional analysis.

### Definition 5

System (4)–(5) is Ulam–Hyers stable if there exists $\lambda =\max ( \lambda _{1},\lambda _{2},\lambda _{3} ) >0$ and $\epsilon =\max ( \epsilon _{1},\epsilon _{1},\epsilon _{1} ) >0$, for each $\widetilde{P},\widetilde{S},\widetilde{I}\in E\times E\times E$, with the following inequalities:

19 $$ \textstyle\begin{cases} \vert {}^{\mathrm{ABC}}\mathbb{D}_{0^{+}}^{\alpha }\widetilde{P}(t)-W_{1}(t,\widetilde{P}) \vert \leq \epsilon _{1}, \\ \vert {}^{\mathrm{ABC}}\mathbb{D}_{0^{+}}^{\alpha }\widetilde{S}(t)-W_{2}(t,\widetilde{S}) \vert \leq \epsilon _{2}, \\ \vert {}^{\mathrm{ABC}}\mathbb{D}_{0^{+}}^{\alpha }\widetilde{I}(t)-W_{3}(t,\widetilde{I}) \vert \leq \epsilon _{3},\end{cases} $$

then there exists $(P,S,I)\in E\times E\times E$ satisfying the coupled system (4) with the following initial conditions:

20 $$ \textstyle\begin{cases} P(0)=\widetilde{P}(0), \\ S(0)=\widetilde{S}(0), \\ I(0)=\widetilde{I}(0),\end{cases} $$

such that

$$ \bigl\Vert ( \widetilde{P},\widetilde{S},\widetilde{I} ) -(P,S,I) \bigr\Vert _{\varOmega }\leq \lambda \epsilon . $$

### Remark 1

Consider a small perturbation $g_{1}\in C[0,T] $ that depends only on the solution such that $g_{1}(0)=0$ with the following properties:

1. $\vert g_{1}(t) \vert \leq \epsilon _{1}$, for $t\in [ 0,T]$ and $\epsilon _{1}>0$.
2. Furthermore, one has
  $$ {}^{\mathrm{ABC}}\mathbb{D}_{0^{+}}^{\alpha }\widetilde{P}(t)=W_{1}(t, \widetilde{P})+g_{1}(t), \quad t\in [0, T]. $$
  Note that we will only discuss the first equation from the proposed system and the rest of the equations are similar in technique, i.e.
  $$ \Vert \widetilde{P}-P \Vert _{E}\leq \lambda _{1} \epsilon _{1}. $$

### Lemma 2

*The solution of the perturbed problem*

21 $$ \textstyle\begin{cases} {}^{\mathrm{ABC}}\mathbb{D}_{0^{+}}^{\alpha }\widetilde{P}(t)=W_{1}(t, \widetilde{P})+g_{1}(t), \\ \widetilde{P}(0)=\widetilde{P}_{0}, \end{cases} $$

*satisfies the relation*

$$ \bigl\vert \widetilde{P}_{g_{1}}(t)-\widetilde{P}(t) \bigr\vert \leq \kappa \epsilon _{1}, $$

*where*$\widetilde{P}_{g_{1}}(t)$*is a solution of* (21), $\widetilde{P}(t)$*satisfies* (19-*a*) *and*$\kappa := ( \frac{\varGamma (\alpha )-\varGamma (\alpha +1)+T^{\alpha }}{\operatorname{ABC}[\alpha ]\varGamma (\alpha )} ) $.

### Proof 3

Thanks to Remark 1, and Lemma 1, the solution of (21) is given by

$$ \widetilde{P}_{g_{1}}(t)= \textstyle\begin{cases} \widetilde{P}_{0}+\frac{1-\alpha }{\operatorname{ABC}[\alpha ]}W_{1}(t,\widetilde{P})+\frac{\alpha }{\operatorname{ABC}[\alpha ]}\frac{1}{\varGamma (\alpha )}\int _{0}^{t}(t-\theta )^{\alpha -1}W_{1}(\theta ,\widetilde{P})\,d\theta \\ \quad{} +\frac{1-\alpha }{\operatorname{ABC}[\alpha ]}g_{1}(t)+ \frac{\alpha }{\operatorname{ABC}[\alpha ]}\frac{1}{\varGamma (\alpha )}\int _{0}^{t}(t-\theta )^{\alpha -1}g_{1}( \theta )\,d\theta .\end{cases}$$

Also, we have

$$ \widetilde{P}(t)=\widetilde{P}_{0}+\frac{1-\alpha }{\operatorname{ABC}[\alpha ]}W_{1}(t,\widetilde{P})+\frac{\alpha }{\operatorname{ABC}[\alpha ]} \frac{1}{\varGamma (\alpha )}\int _{0}^{t}(t-\theta )^{\alpha -1}W_{1}( \theta ,\widetilde{P})\,d \theta . $$

It follows from Remark 1 that

$$\begin{aligned} \bigl\vert \widetilde{P}_{g_{1}}(t)-\widetilde{P}(t) \bigr\vert \leq &\frac{1-\alpha }{\operatorname{ABC}[\alpha ]} \bigl\vert g_{1}(t) \bigr\vert + \frac{\alpha }{\operatorname{ABC}[\alpha ]}\frac{1}{\varGamma (\alpha )} \int _{0}^{t}(t- \theta )^{\alpha -1} \bigl\vert g_{1}(\theta ) \bigr\vert \,d\theta \\ \leq & \biggl( \frac{\varGamma (\alpha )-\varGamma (\alpha +1)+T^{\alpha }}{\operatorname{ABC}[\alpha ]\varGamma (\alpha )} \biggr) \epsilon _{1} \\ =&\kappa \epsilon _{1}. \end{aligned}$$

### Theorem 4

*Under the presumptions of Theorem *3*and condition* (11), *the system* (4)*–*(5) *will be Ulam–Hyers stable in Ω*.

### Proof 4

Let $\widetilde{P}\in E$ be the solution of the inequality (19-a) and the function $P\in E$ be a unique solution of Eq. (4-a) with the condition

22 $$ P(0)=\widetilde{P}(0). $$

That is,

23 $$ P(t)=P_{0}+\frac{1-\alpha }{\operatorname{ABC}[\alpha ]}W_{1}(t,P)+ \frac{\alpha }{\operatorname{ABC}[\alpha ]} \frac{1}{\varGamma (\alpha )} \int _{0}^{t}(t-\theta )^{ \alpha -1}W_{1}( \theta ,P)\,d\theta .$$

Due to (22), $P_{0}=\widetilde{P}_{0}$. Hence Eq. (23) becomes

$$ P(t)=\widetilde{P}_{0}+\frac{1-\alpha }{\operatorname{ABC}[\alpha ]}W_{1}(t,P)+ \frac{\alpha }{\operatorname{ABC}[\alpha ]}\frac{1}{\varGamma (\alpha )} \int _{0}^{t}(t- \theta )^{\alpha -1}W_{1}( \theta ,P)\,d\theta . $$

Thus by condition (11) and Lemma 2, we obtain

$$\begin{aligned} \bigl\vert \widetilde{P}(t)-P(t) \bigr\vert \leq & \bigl\vert \widetilde{P}(t)-\widetilde{P}_{g_{1}}(t) \bigr\vert + \bigl\vert \widetilde{P}_{g_{1}}(t)-P(t) \bigr\vert \\ \leq &\kappa \epsilon _{1}+\frac{1-\alpha }{\operatorname{ABC}[\alpha ]} \bigl\vert W_{1}(t,\widetilde{P})-W_{1}(t,P) \bigr\vert \\ & + \frac{\alpha }{\operatorname{ABC}[\alpha ]}\frac{1}{\varGamma (\alpha )} \int _{0}^{t}(t-\theta )^{\alpha -1} \bigl\vert W_{1}(\theta , \widetilde{P})-W_{1}(\theta ,P) \bigr\vert \,d\theta +\kappa \epsilon _{1} \\ \leq &2\kappa \epsilon _{1}+ \biggl( \frac{1-\alpha }{\operatorname{ABC}[\alpha ]}+ \frac{T^{\alpha }}{\operatorname{ABC}[\alpha ]\varGamma (\alpha )} \biggr) L_{1} \Vert \widetilde{P}-P \Vert , \end{aligned}$$

which implies

$$ \Vert \widetilde{P}-P \Vert _{E}\leq \frac{2\kappa \epsilon _{1}}{1-\varLambda _{1}}, $$

where $\varLambda _{1}= ( \frac{1-\alpha }{\operatorname{ABC}[\alpha ]}+ \frac{T^{\alpha }}{\operatorname{ABC}[\alpha ]\varGamma (\alpha )} ) L_{1}<1$. For $\lambda _{1}=\frac{2\kappa }{1-\varLambda _{1}}$, we get $\Vert \widetilde{P}-P \Vert _{E}\leq \lambda _{1} \epsilon _{1}$.

Similarly, we conclude that $\Vert \widetilde{S}-S \Vert _{E}\leq \lambda _{2} \epsilon _{2}$, and $\Vert \widetilde{I}-I \Vert _{E}\leq \lambda _{3} \epsilon _{3}$, where $\lambda _{\ell }=\frac{2\kappa }{1-\varLambda _{\ell }}$ ($\ell =2,3$). For some $\epsilon ,\lambda >0$,

$$ \bigl\Vert ( \widetilde{P},\widetilde{S},\widetilde{I} ) -(P,S,I) \bigr\Vert _{\varOmega }\leq \lambda \epsilon . $$

Hence the model (4)–(5) is Ulam–Hyers stable.

## Numerical approach

In this part, we give approximation solutions of the ABC fractional model (4)–(5). Then the numerical simulations are acquired via the suggested scheme. To this aim, we employ the modified fractional version for AMB [48] to approximate the fractional integral in the AB sense. To procure an iterative scheme, we go ahead with the first equation of the model (10) as follows:

$$ P(t)-P(0)=\frac{1-\alpha }{\operatorname{ABC}[\alpha ]}W_{1}(t,P)+ \frac{\alpha }{\operatorname{ABC}[\alpha ]} \frac{1}{\varGamma (\alpha )} \int _{0}^{t}(t-\theta )^{ \alpha -1}W_{1}( \theta ,P)\,d\theta . $$

Set $t=t_{n+1}$, for $n=0,1,2,\ldots $ , it follows that

24 $$\begin{aligned}& P(t_{n+1})-P(0) \\& \quad =\frac{1-\alpha }{\operatorname{ABC}[\alpha ]}W_{1}(t_{n},P)+ \frac{\alpha }{\operatorname{ABC}[\alpha ]}\frac{1}{\varGamma (\alpha )}\int _{0}^{t_{n+1}}(t_{n+1}-\theta )^{\alpha -1}W_{1}(\theta ,P)\,d \theta \\& \quad =\frac{1-\alpha }{\operatorname{ABC}[\alpha ]}W_{1}(t_{n},P)+ \frac{\alpha }{\operatorname{ABC}[\alpha ]} \frac{1}{\varGamma (\alpha )}\sum_{\ell =0}^{n} \int _{t_{ \ell }}^{t_{\ell +1}}(t_{n+1}-\theta )^{\alpha -1}W_{1}(\theta ,P)\,d \theta . \end{aligned}$$

Now, we approximate the function $W_{1}(\theta ,P)$ on the interval $[t_{\ell },t_{\ell +1}]$ through the interpolation polynomial as follows:

$$ W_{1} \bigl(\theta ,P(t) \bigr)\cong \frac{W_{1}(t_{\ell },P(t_{\ell }))}{\Delta }(t-t_{\ell -1})+ \frac{W_{1}(t_{\ell -1},P(t_{\ell -1}))}{\Delta }(t-t_{ \ell }), $$

which implies

25 $$\begin{aligned} P(t_{n+1}) =&P(0)+\frac{1-\alpha }{\operatorname{ABC}[\alpha ]}W_{1} \bigl(t_{n},P(t_{n}) \bigr) \\ &{}+\frac{\alpha }{\operatorname{ABC}[\alpha ]}\frac{1}{\varGamma (\alpha )}\sum_{\ell =0}^{n} \biggl( \frac{W_{1}(t_{\ell },P(t_{\ell }))}{\Delta } \int _{t_{\ell }}^{t_{ \ell +1}}(t-t_{\ell -1}) (t_{n+1}-t)^{\alpha -1}\,dt \\ &{} -\frac{W_{1}(t_{\ell -1},P(t_{\ell -1}))}{\Delta } \int _{t_{ \ell }}^{t_{\ell +1}}(t-t_{\ell }) (t_{n+1}-t)^{\alpha -1}\,dt \biggr) \\ =&P(0)+\frac{1-\alpha }{\operatorname{ABC}[\alpha ]}W_{1} \bigl(t_{n},P(t_{n}) \bigr) \\ & {} +\frac{\alpha }{\operatorname{ABC}[\alpha ]}\frac{1}{\varGamma (\alpha )}\sum_{\ell =0}^{n} \biggl( \frac{W_{1}(t_{\ell },P(t_{\ell }))}{\Delta }I_{\ell -1, \alpha }-\frac{W_{1}(t_{\ell -1},P(t_{\ell -1}))}{\Delta }I_{\ell , \alpha } \biggr) . \end{aligned}$$

Now, we compute the integrals $I_{\ell -1,\alpha }$ and $I_{\ell ,\alpha }$ as follows:

$$\begin{aligned} I_{\ell -1,\alpha } =& \int _{t_{\ell }}^{t_{\ell +1}} ( t-t_{ \ell -1} ) ( t_{n+1}-t ) ^{\alpha -1}\,dt \\ =&-\frac{1}{\alpha } \bigl[ ( t_{\ell +1}-t_{\ell -1} ) ( t_{n+1}-t_{\ell +1} ) ^{\alpha }- ( t_{\ell }-t_{ \ell -1} ) ( t_{n+1}-t_{\ell } ) ^{\alpha } \bigr] \\ & {} -\frac{1}{\alpha ( \alpha +1 ) } \bigl[ ( t_{n+1}-t_{ \ell +1} ) ^{\alpha +1}- ( t_{n+1}-t_{\ell } ) ^{ \alpha +1} \bigr] \end{aligned}$$

and

$$\begin{aligned} I_{\ell ,\alpha } =& \int _{t_{\ell }}^{t_{\ell +1}} ( t-t_{ \ell } ) ( t_{n+1}-t ) ^{\alpha -1}\,dt \\ =&-\frac{1}{\alpha } \bigl[ ( t_{\ell +1}-t_{\ell } ) ( t_{n+1}-t_{\ell +1} ) ^{\alpha } \bigr] \\ &{}-\frac{1}{\alpha ( \alpha +1 ) } \bigl[ ( t_{n+1}-t_{ \ell +1} ) ^{\alpha +1}- ( t_{n+1}-t_{\ell } ) ^{ \alpha +1} \bigr]. \end{aligned}$$

Put $t_{\ell }=\ell \Delta $, we get

26 $$\begin{aligned} I_{\ell -1,\alpha } =&-\frac{\Delta ^{\alpha +1}}{\alpha } \bigl[ \bigl( \ell +1- ( \ell -1 ) \bigr) \bigl( n+1- ( \ell +1 ) \bigr) ^{\alpha }- \bigl( \ell - ( \ell -1 ) \bigr) ( n+1-\ell ) ^{\alpha } \bigr] \\ & {} -\frac{\Delta ^{\alpha +1}}{\alpha ( \alpha +1 ) } \bigl[ \bigl( n+1- ( \ell +1 ) \bigr) ^{\alpha +1}- ( n+1-\ell ) ^{\alpha +1} \bigr] \\ =&\frac{\Delta ^{\alpha +1}}{\alpha ( \alpha +1 ) } \bigl[ -2 ( \alpha +1 ) ( n-\ell ) ^{\alpha }+ ( \alpha +1 ) ( n+1-\ell ) ^{\alpha }- ( n-\ell ) ^{\alpha +1}+ ( n+1-\ell ) ^{\alpha +1} \bigr] \\ =&\frac{\Delta ^{\alpha +1}}{\alpha ( \alpha +1 ) } \bigl[ ( n-\ell ) ^{\alpha } \bigl( -2 ( \alpha +1 ) - ( n-\ell ) \bigr) + ( n+1-\ell ) ^{ \alpha } ( \alpha +1+n+1-\ell ) \bigr] \\ =&\frac{\Delta ^{\alpha +1}}{\alpha ( \alpha +1 ) } \bigl[ ( n+1-\ell ) ^{\alpha } ( n-\ell +2+\alpha ) - ( n-\ell ) ^{\alpha } ( n-\ell +2+2 \alpha ) \bigr] \end{aligned}$$

and

27 $$\begin{aligned} I_{\ell ,\alpha } =&-\frac{\Delta ^{\alpha +1}}{\alpha } \bigl[ ( \ell +1-\ell ) \bigl( n+1- ( \ell +1 ) \bigr) ^{\alpha } \bigr] \\ &{}-\frac{h^{\alpha +1}}{\alpha ( \alpha +1 ) } \bigl[ \bigl( n+1- ( \ell +1 ) \bigr) ^{\alpha +1}- ( n+1-\ell ) ^{\alpha +1} \bigr] \\ =&\frac{\Delta ^{\alpha +1}}{\alpha ( \alpha +1 ) } \bigl[ - ( \alpha +1 ) ( n-\ell ) ^{\alpha }- ( n-\ell ) ^{\alpha +1}+ ( n+1-\ell ) ^{ \alpha +1} \bigr] \\ =&\frac{\Delta ^{\alpha +1}}{\alpha ( \alpha +1 ) } \bigl[ ( n-\ell ) ^{\alpha } \bigl( - ( \alpha +1 ) - ( n-\ell ) \bigr) + ( n+1-\ell ) ^{ \alpha +1} \bigr] \\ =&\frac{\Delta ^{\alpha +1}}{\alpha ( \alpha +1 ) } \bigl[ ( n+1-\ell ) ^{\alpha +1}- ( n-\ell ) ^{\alpha } ( n-\ell +1+\alpha ) \bigr] . \end{aligned}$$

Substituting (26) and (27) into (25), we get

28 $$\begin{aligned} P(t_{n+1}) =&P(t_{0})+\frac{1-\alpha }{\operatorname{ABC}[\alpha ]}W_{1} \bigl(t_{n},P(t_{n}) \bigr) \\ &{} + \frac{\alpha }{\operatorname{ABC}[\alpha ]}\sum_{\ell =0}^{n} \biggl( \frac{W_{1}(t_{\ell },P(t_{\ell }))}{\varGamma (\alpha +2)} \Delta ^{\alpha } \bigl[ (n+1-\ell )^{\alpha }(n-\ell +2+\alpha ) \\ &{}-(n- \ell )^{\alpha }(n-\ell +2+2\alpha ) \bigr] \\ &{}- \frac{W_{1}(t_{\ell -1},P(t_{\ell -1}))}{\varGamma (\alpha +2)}\Delta ^{\alpha } \bigl[ (n+1-\ell )^{\alpha +1}-(n-\ell )^{\alpha }(n- \ell +1+\alpha ) \bigr] \biggr) . \end{aligned}$$

Similarly

29 $$\begin{aligned} S(t_{n+1}) =&S(t_{0})+\frac{1-\alpha }{\operatorname{ABC}[\alpha ]}W_{2} \bigl(t_{n},S(t_{n}) \bigr) \\ &{} + \frac{\alpha }{\operatorname{ABC}[\alpha ]}\sum_{\ell =0}^{n} \biggl( \frac{W_{2}(t_{\ell },S(t_{\ell }))}{\varGamma (\alpha +2)} \Delta ^{\alpha } \bigl[ (n+1-\ell )^{\alpha }(n-\ell +2+\alpha ) \\ &{}-(n- \ell )^{\alpha }(n-\ell +2+2\alpha ) \bigr] \\ &{}- \frac{W_{2}(t_{\ell -1},S(t_{\ell -1}))}{\varGamma (\alpha +2)}\Delta ^{\alpha } \bigl[ (n+1-\ell )^{\alpha +1} -(n-\ell )^{\alpha }(n- \ell +1+\alpha ) \bigr] \biggr) \end{aligned}$$

and

30 $$\begin{aligned} I(t_{n+1}) =&I(t_{0})+\frac{1-\alpha }{\operatorname{ABC}[\alpha ]}W_{3} \bigl(t_{n},I(t_{n}) \bigr) \\ &{} +\frac{\alpha }{\operatorname{ABC}[\alpha ]}\sum_{\ell =0}^{n} \biggl( \frac{W_{3}(t_{\ell },I(t_{\ell }))}{\varGamma (\alpha +2)} \Delta ^{\alpha } \bigl[ (n+1-\ell )^{\alpha }(n-\ell +2+\alpha ) \\ &{}-(n- \ell )^{\alpha }(n-\ell +2+2\alpha ) \bigr] \\ &{}- \frac{W_{3}(t_{\ell -1},I(t_{\ell -1}))}{\varGamma (\alpha +2)}\Delta ^{\alpha } \bigl[ (n+1-\ell )^{\alpha +1} -(n-\ell )^{\alpha }(n- \ell +1+\alpha ) \bigr] \biggr) . \end{aligned}$$

### Numerical interpretation and discussion

**Figure 1:**
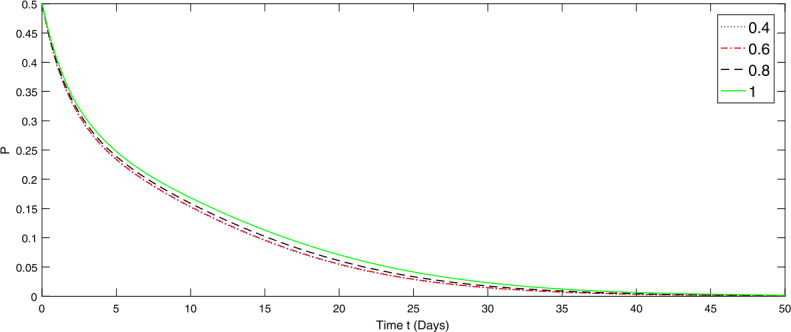
Graphical representation of numerical solution for specie *P* at various fractional orders of the considered model (4)

**Figure 2:**
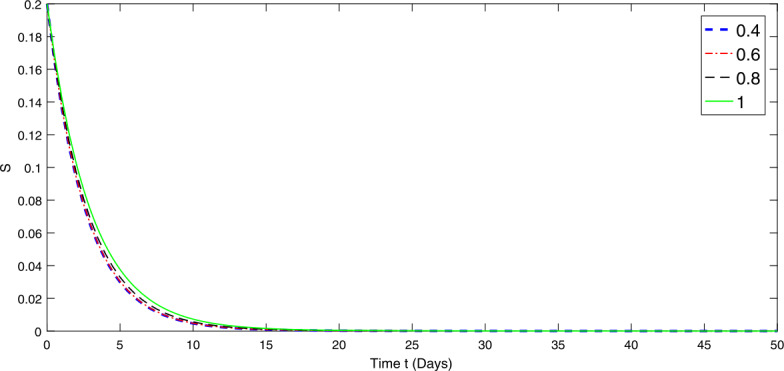
Graphical representation of numerical solution for specie *S* at various fractional orders of the considered model (4)

**Figure 3:**
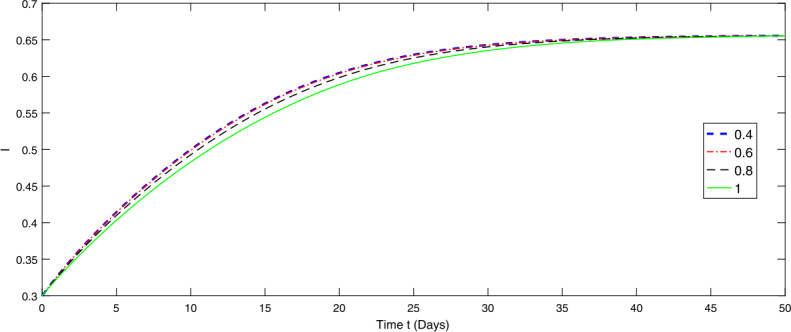
Graphical representation of numerical solution for specie *I* at various fractional orders of the considered model (4)

Now, to present the numerical simulations of the ABC fractional model (4)–(5), we apply the iterative solution contained in (28)–(30). Take the time range up to 100 units. The numerical values of the parameters applied in the simulations are specified in Table 1. The graphical representations of numerical solution for species *P*, *S*, *I* at various fractional orders, $\alpha =0.4,0.6,0.8,1.0$, of the considered model (4) are given in Figs. 1–3, respectively.

**Table 1:** The physical interpretation of the parameters and numerical values

Parameters	Physical description	Numerical value
$P_{0}$	initial population density of prey	0.5
$S_{0}$	initial population density of susceptible predator	0.3
$I_{0}$	initial population density infected predator	0.2
*a*	saturation constant while susceptible predators attack the prey	0.00073
*b*	search rate of the prey toward susceptible predator	0.0001
*c*	conversion rate of susceptible predator due to prey	0.0003
*d*	disease transmission coefficient	0.007
*k*	carrying capacities of prey population	0.003
$b_{1}$	proportionality constant	0.004
$r_{1}$	growth rate of prey population	0.0003
*m*	death rate of susceptible predator	0.004
*n*	death rate of infected predator	0.003

From Figs. 1–3, we observe that species *I* depends on species *P* and *S*. Therefore the papulation density of specie *P* and *S* gradually go on decreasing with different rate due to the fractional order in the first 50 days. The lower the fractional order, the faster the decay rate and hence the more rapidly the system becomes stable and vice versa. On the other hand, the species *I* is going on increasing with different rate, the lower the order the slower is the growth rate until it becomes stable and vice versa. The fractional order greatly affects the stability of the system and also provides the global nature of the dynamics of the considered model.

Here we claim that the established numerical technique is powerful and converges for the ABC fractional derivative. Meanwhile the iterative techniques like perturbation and decomposition methods do not show the perfect behavior for the said derivatives for approximate solutions in many cases.

## Conclusion

In this paper, the population density model of prey and sensitive predatory and infected predatory has been studied theoretically and numerically. Theoretically, the existence and stability results in the sense of Ulam–Hyers have been obtained through the help of fixed point theory and nonlinear analysis. Numerically, the approximation solution of the ABC fractional model (4) has been discussed via the use of a fractional Adam Bashforth method. Moreover, the behavior of the solutions of the model (4) has also been explained through graphs using some numerical values for the parameter. The obtained results play an important role in developing the theory of fractional analytical dynamics of various phenomena of real-world problems.
